# Triple transitions and QRS alternans with left bundle branch area pacing: Insights into conduction system pacing physiology

**DOI:** 10.1016/j.hrcr.2025.05.021

**Published:** 2025-05-30

**Authors:** Julia Herbert, Haran Burri

**Affiliations:** Cardiac Pacing Unit, Department of Cardiology, University Hospital of Geneva, Geneva, Switzerland

**Keywords:** Conduction system pacing, Left bundle branch pacing, Electrocardiography, Electrogram, Conduction system

**Key Teaching Points**

• Implantation of a left bundle branch area pacing (LBBAP) lead at a branching point of the left-bundle branch can lead to multiple QRS morphologies, related to capture of the myocardium and of one or the other (or both) fascicles.
• V6 R-wave peak time (V_6_RWPT) during LBBAP with conduction system capture may be longer than His-V_6_RWPT if the fascicle being captured has slow conduction or a long pathway to reach V6.
• Variable splitting of the electrogram may be observed with selective fascicular capture, which we hypothesize will depend on the distance to or conduction delay from the exit site of the fascicle.
• QRS alternans may be observed with LBBAP at high pacing rates, most probably owing to differences in refractory periods between fascicles. This phenomenon may be output dependent, owing to pacing in the relative refractory period.

## Introduction

Left bundle branch area pacing (LBBAP) is being increasingly used as an alternative to right ventricular and biventricular pacing.^1^, ^2^, ^3^, ^4^ Conduction system capture is identified by transitions in paced QRS morphology during threshold tests and a number of electrocardiographic criteria such as V6 R-wave peak time (V_6_RWPT) and V6–V1 interpeak interval.^5^, ^6^, ^7^, ^8^, ^9^ The gold standard of these criteria at implantation is a transition in QRS morphology with decrementing output during unipolar pacing, with loss of myocardial capture or conduction system capture (resulting respectively in selective conduction system pacing or myocardial capture only). In these instances, 2 distinct electrocardiographic morphologies are observed. We describe a case of LBBAP that shows 4 different QRS morphologies during threshold testing and alternans of paced QRS morphology and provide an explanation for these findings.

## Case report

**Figure 1:**
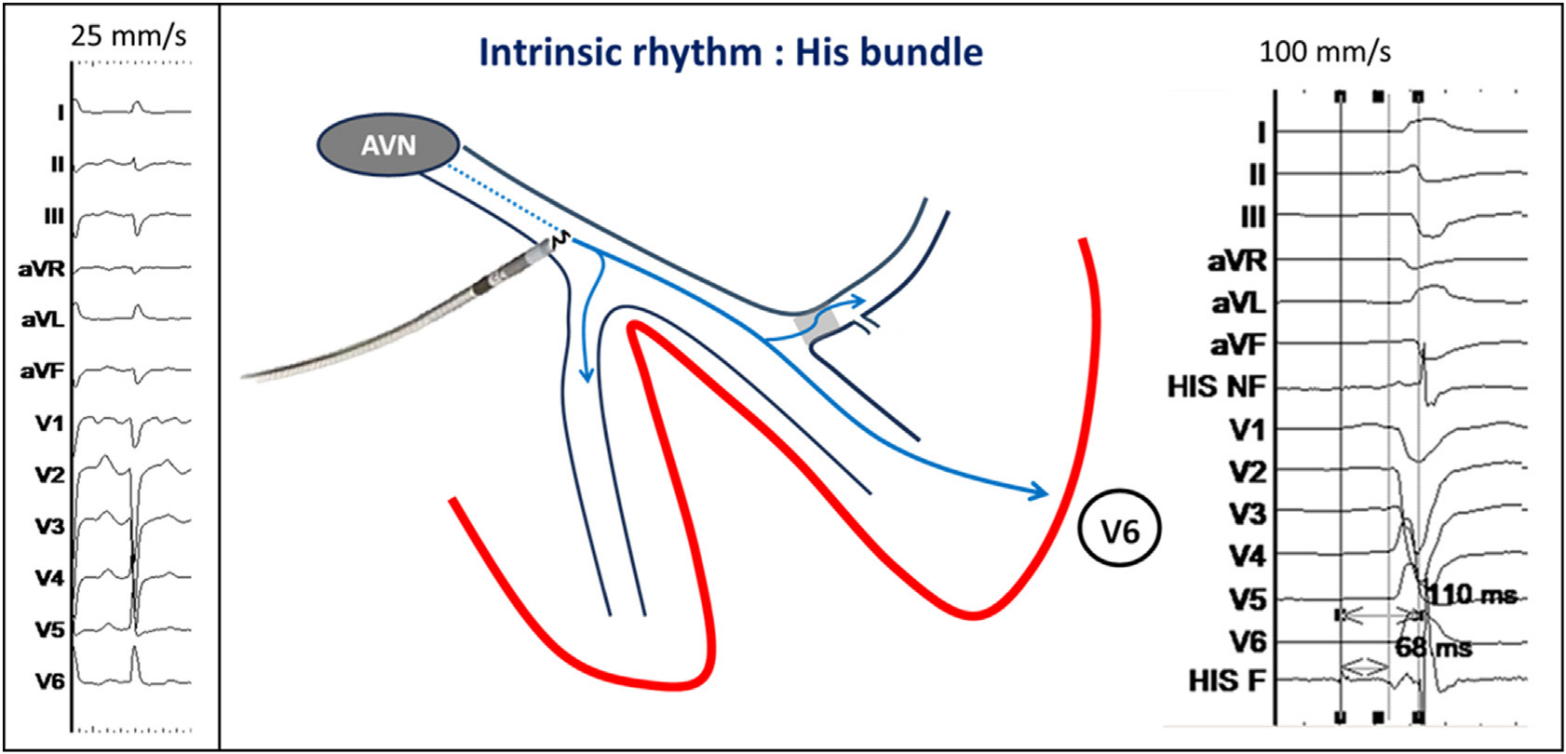
Intrinsic rhythm in atrial fibrillation with QRS of 120 ms and leftward axis deviation (−39°) indicating delayed conduction in the left anterior fascicle. The right panel shows recording of a His potential by the pacing lead with His-R-wave peak time in V6 of 110 ms and His-ventricular interval of 68 ms. HIS F = His filtered (30–500 Hz); HIS NF = His nonfiltered (0.5–500 Hz).

A 67-year-old male patient with rapidly conducted permanent atrial fibrillation and nonischemic cardiomyopathy and an ejection fraction of 25% underwent implantation of an implantable cardioverter-defibrillator to be followed by atrioventricular node ablation. The baseline QRS showed intraventricular conduction delay with a QRS of 122 ms and leftward axis of −39° (see Figure 1).

Given that all LBBAP implantations at our center are performed with simultaneous video recordings of the fluoroscopy, electrophysiology recording system, pacing system analyzer screens, and a camera focused on the operating site, the procedure could be accurately reconstituted.

**Figure 2:**
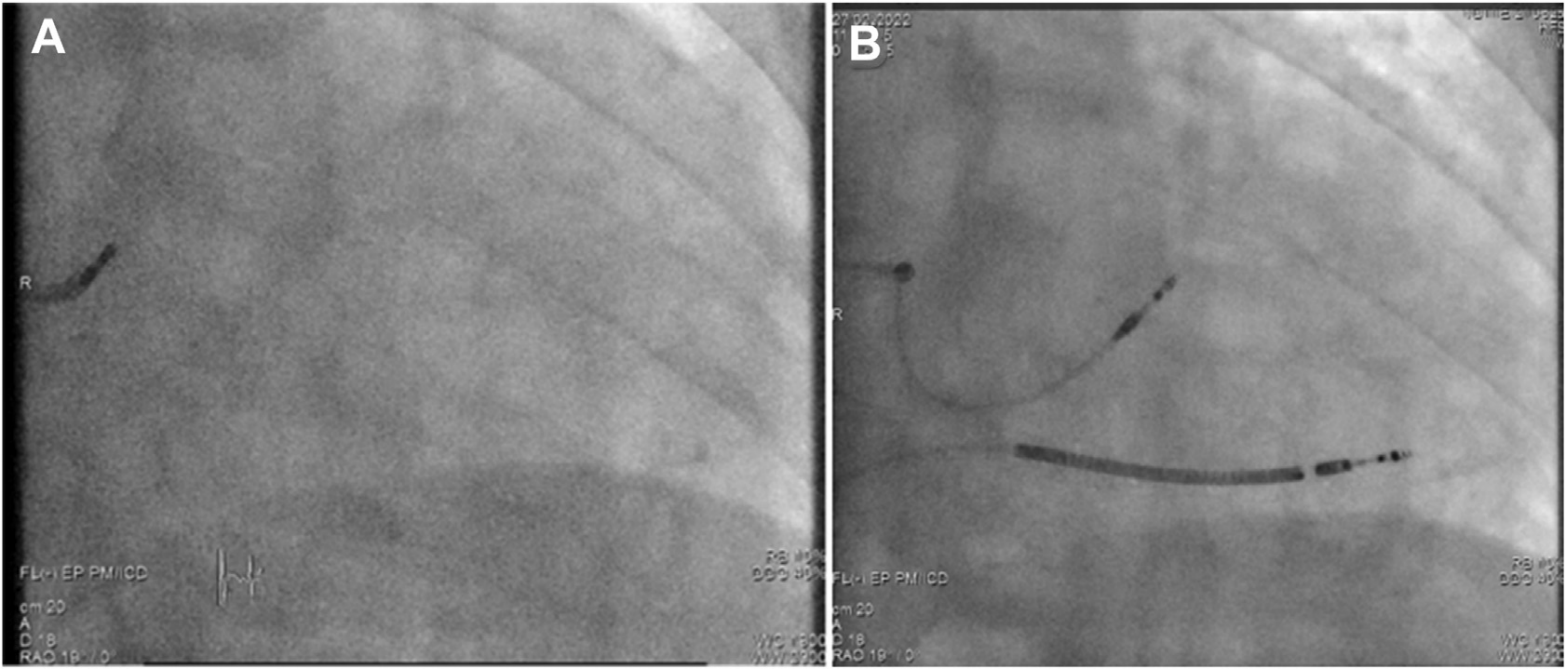
Fluoroscopic images during implantation in right anterior oblique 19° projection. **A:** The His bundle location. **B:** The final lead position.

**Figure 3:**
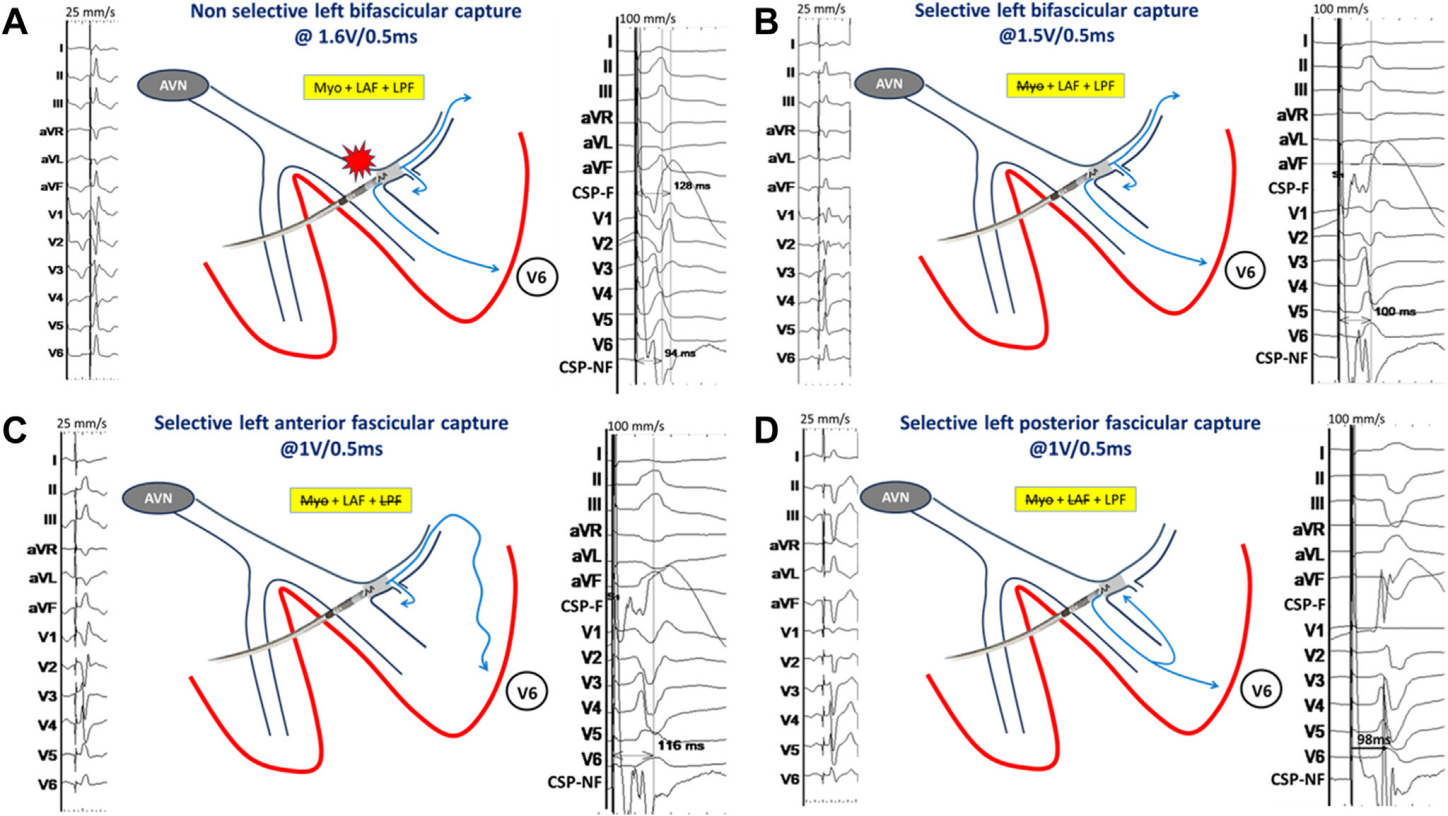
Four QRS morphologies observed at left bundle branch area pacing implantation during threshold testing with corresponding measurement of V6 R-wave peak time (V_6_RWPT). The site of conduction block is depicted by the *gray zone*. **A:** Nonselective bifascicular capture. **B:** Selective bifascicular capture (note splitting of the ventricular electrogram). **C:** Selective left anterior fascicular capture (note increasing positivity in the inferior leads). Splitting of the ventricular electrogram is similar to that during selective bifascicular capture, presumably owing to the relatively proximal conduction system exit site. V_6_RWPT is delayed, presumably owing to a longer activation pathway to the V6 electrode. **D:** Selective left posterior fascicular capture (note superior axis). Markedly increased splitting of the electrogram is presumably caused by the more distal conduction system exit site. The V_6_RWPT is comparable with nonselective and selective bifascicular capture, implying that the activation pathway to the V6 electrode is predominantly affected by conduction via the posterior fascicle. CSP F = conduction system pacing filtered (30–500 Hz); CSP NF = conduction system pacing nonfiltered (0.5–500 Hz); LAF = left anterior fascicle; LPF = left posterior fascicle; Myo = myocardial.

**Figure 4:**
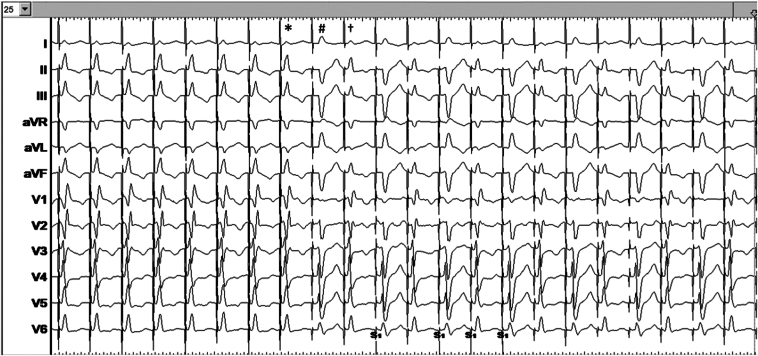
Electrocardiogram showing transition from nonselective bifascicular capture at output of 2 V/0.5 ms (∗) to alternans between selective posterior fascicular capture (#) and selective bifascicular capture (†) at 1.0 V/0.5 ms during pacing at 140 beats per minute. Sweep speed is 25 mm/s.

An Abbott Tendril STS lead (Abbott, Sylmar, CA) was initially positioned on the His bundle to provide a fluoroscopic landmark, and for evaluating conduction physiology, a Biotronik (Berlin, Germany) Selectra 3-dimensional 55 delivery catheter was used. The His-ventricular delay was measured at 68 ms and the His-R-wave peak time in V6 (His-V_6_RWPT) at 110 ms (Figure 1). The catheter was advanced into the right ventricle, and the lead was deployed into the septum with rotations of the lead body during unipolar pacing delivered via the stylet. A fascicular potential was not observed at the final site. Threshold testing performed at 130 beats per minute (bpm) demonstrated transition from nonselective capture to selective conduction system capture at 2 V/0.5 ms and loss of capture at 0.6 V/0.5 ms without any further transitions (tracings not shown). After implantation of the LBBAP lead, an implantable cardioverter-defibrillator lead was implanted in the right ventricular apex (Figure 2). Threshold testing of the LBBAP lead was again performed 15 minutes after initial testing, at a rate of 140 bpm, revealing this time multiple transitions in QRS morphology, which are summarized along with their corresponding electrical measurements in Figure 3. We also observed QRS alternans during pacing at 1 V/0.5 ms (which was absent at 2 V/0.5 ms) (see Figure 4). Selective capture of the anterior fascicle was lost at 22 minutes after initial testing, with transition from selective bifascicular capture to selective posterior fascicular capture (the threshold of which was 0.9 V/0.5 ms).

## Discussion

This case illustrates different interesting findings: (1) As many as 4 different QRS morphologies may be obtained at different pacing outputs with unipolar LBBAP owing to capture of different tissues, (2) LBBAP-V_6_RWPT with conduction system capture can be longer than His-V_6_RWPT, (3) splitting of the electrogram during selective fascicular capture can be variable, and (4) QRS alternans may be transiently observed with LBBAP.

### Triple transition during decrementing unipolar pacing output, with 4 QRS morphologies

The QRS morphologies were indicative of nonselective bifascicular capture, selective bifascicular capture, selective anterior (also termed “superior”) fascicular capture, and selective posterior (also termed “inferior”) fascicular capture at different pacing outputs. This suggests that the LBBAP lead was positioned at a branching site of the left-sided conduction system. It is recognized that ramification of the left bundle is usually a trifurcation, which includes a septal fascicle in addition to the anterior and posterior fascicles.^10^ It is unclear in the present case whether the septal fascicle was captured along with the anterior or the posterior fascicle. A recent report has also described cases with triple transitions (ie, 4 paced QRS morphologies) with unipolar LBBAP pacing, attributed to the same mechanism as in our patient.^11^ A previous case report described 3 paced QRS morphologies with threshold testing of LBBAP,^12^ interpreted as being caused by capture of septal myocardium, left posterior fascicle, and the Purkinje network, but transitions in QRS morphology were less pronounced than in our case.

### V_6_RWPT during LBBAP with conduction system capture may be longer than His-V_6_RWPT

Given that the LBBAP lead is positioned distal to the His bundle, one would expect the V_6_RWP to be *shorter* than the His-ventricular interval in the presence of conduction system capture. In the present case, we observed slightly *longer* V_6_RWPT during selective left anterior fascicular capture than His-V_6_RWPT (116 ms vs 110 ms). We hypothesized that selective capture of the anterior fascicle resulted in slower conduction owing to underlying disease (as suggested by the baseline left axis deviation) and/or a longer activation pathway to reach the lateral left ventricle. The region underlying the V_6_ electrode was most probably preferentially activated by the posterior fascicle (explaining shorter V_6_RWPT during LBBAP with capture of the posterior fascicle during bifascicular and selective posterior fascicular capture, which were shorter than the His-V_6_RWPT).

### Variable splitting of the electrogram with selective fascicular capture

Splitting of the local ventricular electrogram during LBBAP threshold testing is a well-recognized sign of transition from nonselective to selective conduction system capture.^5^ Our case illustrates variable splitting of the ventricular electrogram (most pronounced during selective posterior fascicular capture). This phenomenon may be explained by a more distal exit site of the Purkinje fibers from the posterior fascicle than the anterior fascicle. Alternatively, slower conduction from the exit site toward the site of implantation of the LBBAP lead (eg, owing to scar tissue) may explain the greater delay to the local ventricular electrogram.

### QRS alternans

This observation, to the best of our knowledge, is described for the first time with LBBAP in this case report. QRS alternans may be explained by a longer refractory period of the left anterior fascicle (eg, owing to underlying conduction disease or trauma by the lead screw), leading to an alternans between bifascicular capture and capture of the left posterior fascicle only, at a constant pacing output and at the relatively rapid rate of 140 bpm (which was not initially observed during pacing at 130 bpm—which supports our hypothesis). The phenomenon was also output dependent, which may be explained by capture of the anterior fascicle during its relative refractory period at a higher pacing output. Another hypothesis is that beat-to-beat variation in lead orientation and contact with the conduction tissue led to alternating preferential capture of the posterior fascicle, which is less likely.

## Conclusion

Our case illustrates a number of unique findings related to LBBAP. Careful analysis of surface and endocavitary tracings presents us with a golden opportunity to study conduction system physiology.

## Disclosures

H.B. has received speaker fees and institutional research grants from Abbott, Biotronik, Boston Scientific, Medtronic, and MicroPort. The other author has no conflicts of interest to disclose.
